# Supplementary material for: MixOmics Integration of Biological Datasets Identifies Highly Correlated Variables of COVID-19 Severity
Source: Int J Mol Sci. 2025 May 15;26(10):4743. doi: 10.3390/ijms26104743 (PMC12111767; doi:10.3390/ijms26104743)
Supplement: Supplementary file 1 [file ijms-26-04743-s001.zip › ijms-3410327-supplementary/2025_R1_IJMS_Mixomics _Supplement/2024_IJMS_Mixomics _Supplement.pdf]

SUPPLEMENRATY INFORMATION

Title

MixOmics Integration of Biological Datasets Identifies Highly Correlated Key Variables of COVID-19 severity.

Authors

Noa C. Harriott[1,2,3*], Michael S. Chimenti[4*], Gregory Bonde[1], Amy L. Ryan[1,2,3#]

Affiliations

[1]Department of Anatomy and Cell Biology, Carver College of Medicine, University of Iowa, Iowa City IA 52240

[2]Department of Stem Cell Biology and Regenerative Medicine, University of Southern California, Los Angeles CA 90033

[3]Hastings Center for Pulmonary Research, Division of Pulmonary, Critical Care and Sleep Medicine, Department of Medicine, University of Southern California, Los Angeles CA 90033

[4]Iowa Institute of Human Genetics, Carver College of Medicine, University of Iowa, Iowa City IA 52240

*Both NCH and MSC contributed equally to this work.

#Corresponding Author

Amy L. Ryan, PhD

Associate Professor: Anatomy and Cell Biology

Associate Director: Center for Gene Therapy

BSB, 1-400 Core

University of Iowa

51 Newton Road

Iowa City, Iowa 52241

Email: amy-l-ryan@uiowa.edu

Tel: 319 335 8908

Conflict of Interest Statement

The authors have declared that no conflict-of-interest exists.

Supplementary Figures

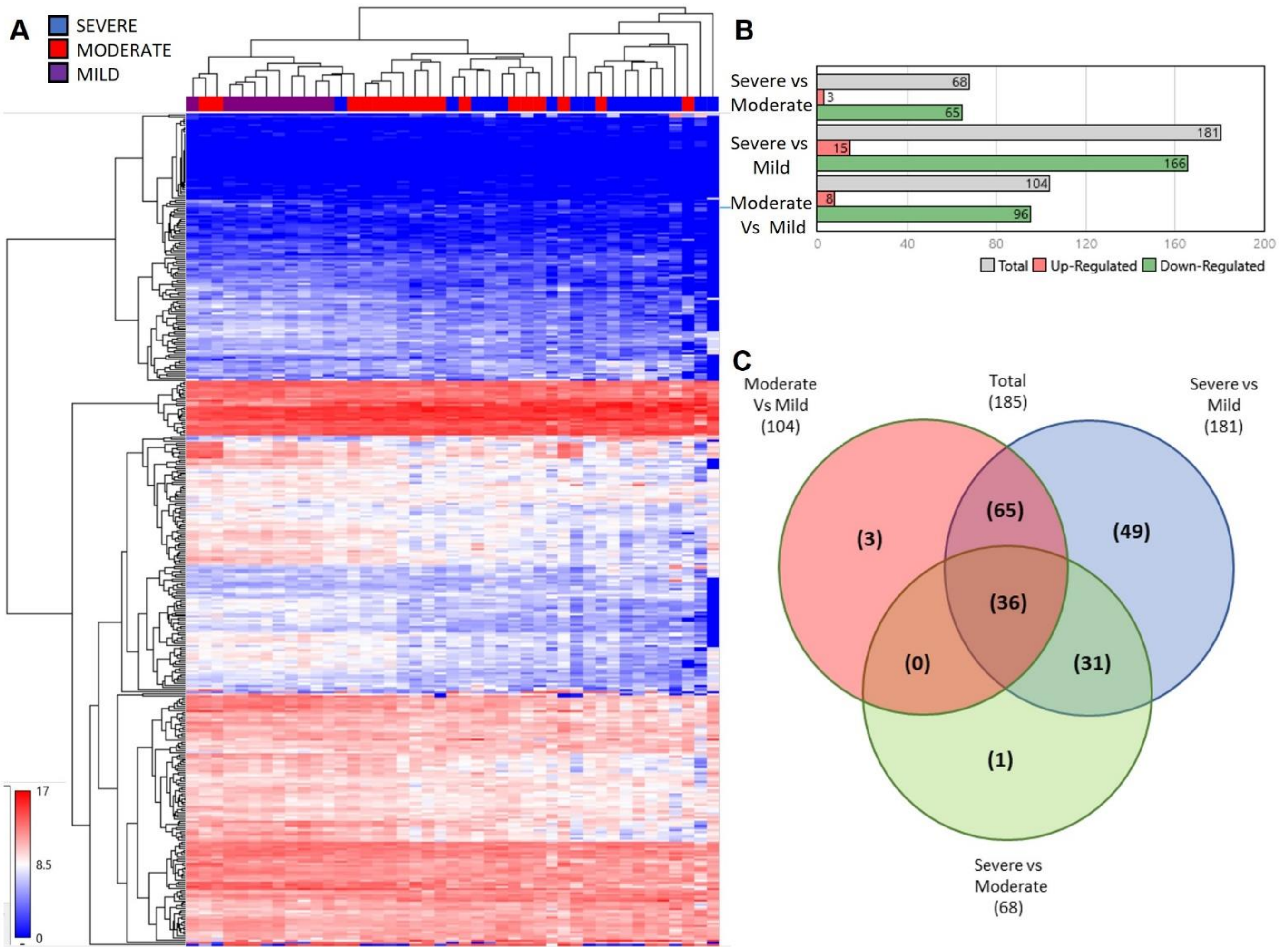

Supplementary Figure S1. DEG between COVID-19 cohorts. A) Unsupervised clustering of gene expression in a heatmap with cohorts identified as severe (blue) moderate (red) and mild (purple). **B)** Table of DEG between the paired cohort comparisons identified. Total DEG (grey), upregulated DEG (red) and down regulated DEG (green). **C)** Venn Diagram showing the overlap of DEG between cohorts. 36 genes of a total of 185 DEG were differentially expressed across all cohorts.

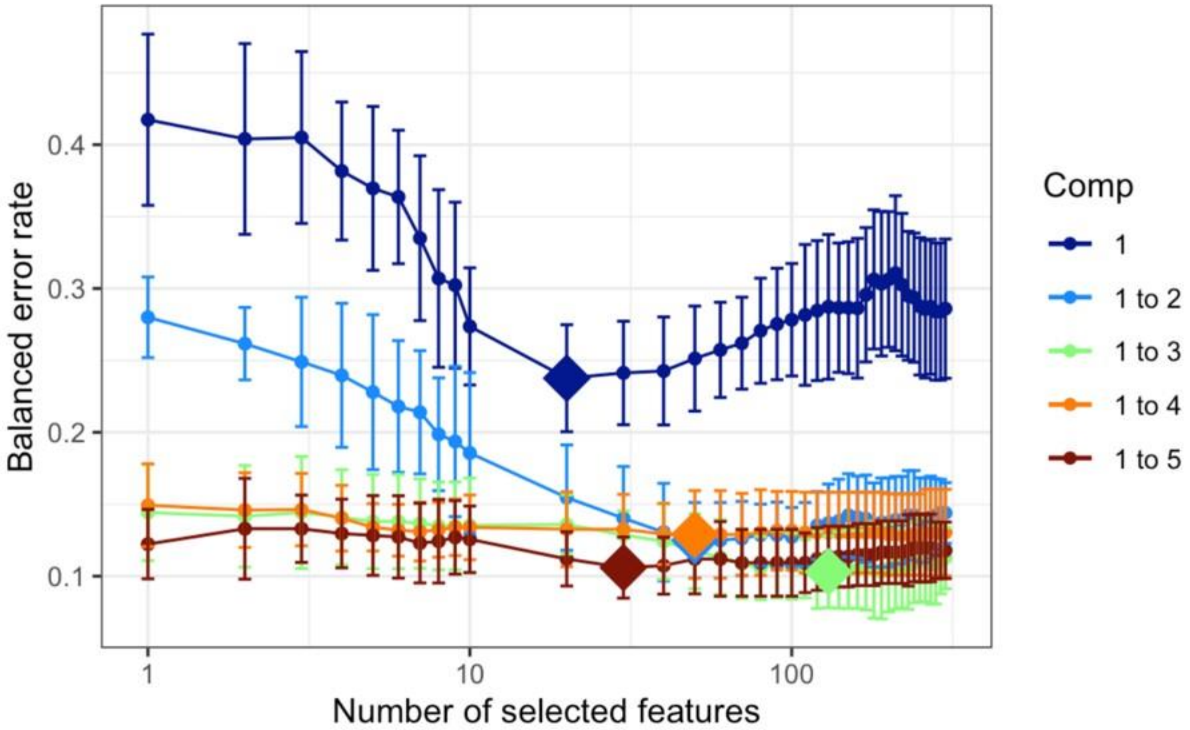

Supplementary Figure S2. sPLA-DA feature selection tuning of the transcriptomics dataset. 5-fold cross-validation of the sPLS-DA RNA-seq model with 10 repeats using a “balanced error rate (BER).” Plotting the BER as a function of the number of features selected showed that two components (light blue) performed nearly as well as 3-5 components for certain values of the “keepX” parameter.

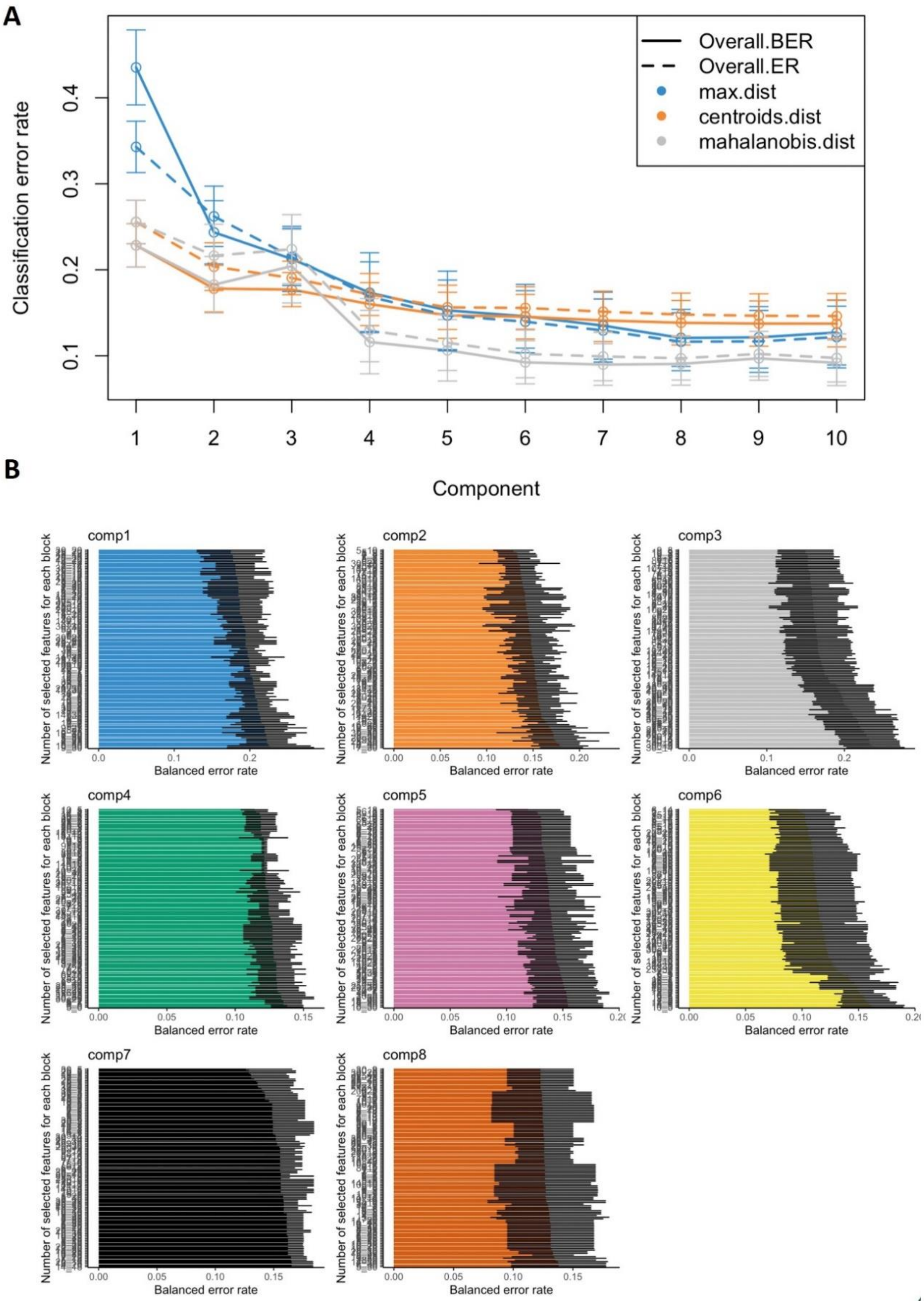

Supplementary Figure S3. DIABLO model performance feature selection. A) Performance testing with K-fold cross validation (K=5) and 50 repeats showed that the overall balanced error rate (BER) **decreased with** each component until leveling out around 8 components. **B**) Feature selection tuning on 8 components of the DIABLO model.

Supplementary Tables

Supplementary Table S1: Top 117 features selected by the DIABLO model

The top 117 features selected by the DIABLO model	
**RNA**	**PROTEIN**
RB1	IL-17C
CYBB	IL-2RB
VEGFA	Flt3L
ZEB1	ADA
CD68	CRNN
TNFRSF9	VEGFR-3
IL18	IL-10RA
FOXO1	LIF-R
GBP1	NGF
IDO1	TNFB
ID2	MICA
IL3RA	MICB
ITGB1	CD5
PSMB9	LYPD3
PTPN11	SPARC
CCL5	CD70
TBP	GZMB
SKAP2	CDCP1
CTSS	EN-RAGE
GZMB	SYND1
GRAP2	HGF
NKG7	WFDC2
PVR	
CD226	
DDX58	
BRCA2	
C1QB	
IL10	
IL12A	
CD53	
CCR2	
TNFRSF17	
CD38	
CD52	
ITGAE	
MAPK1	
CDKN3	
IKZF1	
TNFAIP8	
SAMHD1	
TLR7	
HLA-DRA	

SLAMF7	
HAVCR2	
BTLA	
IL15	
SELL	
TFRC	
CD19	
FCRLA	
CXCR2	
TNFRSF9	
AIF1	
CD79A	
HLA-DQA1	
MIF	
IRF4	
NT5E	
IFITM1	
TNFSF10	
CTSS	
CCR4	
IRS1	
IGSF6	
IFITM2	
TNFSF13B	
KIR2DL1	
KREMEN1	
HLA-DPA1	
LMNA	
ARG1	
CD4	
CA4	
IL2RB	
ZAP70	
IL10RA	
CCR7	
MYC	
TCF7	
CSF1R	
ITK	
CD6	
SIT100	
CD8B	

Supplementary Databases

Supplementary Database S1: DEG between cohorts for Day 1 samples only.

Supplementary Database S2: Top GO and Kegg Pathway Functional enrichments in the top features contributing to the classification performance of the final DIABLO model along the first component.

Supplementary Database S3: Top GO Pathway Functional enrichments in the top features contributing to the classification performance of the final DIABLO model along the second component.
